# The HBZ gene, a key player in HTLV-1 pathogenesis

**DOI:** 10.1186/1742-4690-6-71

**Published:** 2009-08-03

**Authors:** Masao Matsuoka, Patrick L Green

**Affiliations:** 1 Laboratory of Virus Control, Institute for Virus Research, Kyoto University, Kyoto 606-8507, Japan; 2 Center for Retrovirus Research, Departments of Veterinary Biosciences and Molecular Virology, and Medical Genetics, Comprehensive Cancer Center and Solove Research Institute, The Ohio State University, Columbus, OH 43210, USA

## Abstract

Human T-cell leukemia virus type 1 (HTLV-1) causes adult T-cell leukemia/lymphoma (ATL) and is also associated with a variety of lymphocyte-mediated diseases. The *HTLV-1 basic leucine zipper *(*HBZ*) gene, found to be consistently expressed in ATL, has recently been the subject of intensive research efforts. In this review, we summarize recent findings about HBZ and discuss its roles and functions not only in the virus life cycle, but also in HTLV-1 disease pathogenesis.

## Background

Adult T-cell leukemia/lymphoma (ATL) was proposed as a distinct clinical entity in 1975 by Takatsuki et al. [1]. An etiological linkage between ATL and virus infection was suggested by the geographical clustering of ATL patients in southwestern Japan. Subsequently, human T-cell leukemia virus type 1 (HTLV-1) was discovered in 1980 as the cause of ATL and was the first retrovirus associated with a disease in humans [2,3]. Early focus on the mechanism of cell transformation has been on the *trans*-acting viral regulatory protein Tax. Although studied extensively, the role of *tax *in HTLV-1 leukemogenesis remains unclear since expression of the *tax *gene as well as other viral genes are not always detected in ATL cells [4]. More recently, expression of the *HTLV-1 bZIP factor gene *(*HBZ*), an antisense mRNA transcribed from the 3' LTR, has been shown to be consistently expressed in ATL cells [5]; thus, HBZ may have a functional role in cellular transformation and leukemogenesis.

### Expression of *HBZ *genes in ATL cells and T-cells from asymptomatic carriers

Among the HTLV-1 regulatory and accessory genes, the *tax *gene is thought to play a central role in leukemogenesis since it immortalizes T-lymphocytes *in vitro*, and induces various cancers in transgenic animals [6,7]. However, an enigma is that Tax expression is not detected in about 60% of leukemia cases [4]. Three mechanisms for inactivating Tax expression in ATL cells have been described: 1) genetic changes (nonsense mutation, deletion, and insertion) of the *tax *gene [4,8], 2) deletion of the 5' long terminal repeat (LTR) containing the viral promoter [9,10], and 3) DNA methylation of the 5 'LTR leading to promoter inactivation [11,12]. One possible scenario is that since Tax is the major target of cytotoxic T-lymphocytes (CTL) *in vivo *[13], these mechanisms to disrupt or decrease Tax expression facilitate the escape of ATL cells from host CTL. Interestingly, analyses of HTLV-1 provirus in ATL cells showed that the 3' LTR was not deleted and remained unmethylated. In addition, the pX region (located between *env *and the 3' LTR) encoding the regulatory and accessory genes also is maintained. Detailed analyses of defective proviruses lacking the 5' LTR revealed that all cases maintained the *HBZ *gene and the 3' LTR. In one ATL case, the polyadenylation site of the *HBZ *gene was deleted [10]; however, this *HBZ *gene utilized a downstream cellular polyadenylation signal for transcription. Taken together, these findings suggest that *HBZ *gene transcription is indispensable for the development of ATL.

**Figure 1:**
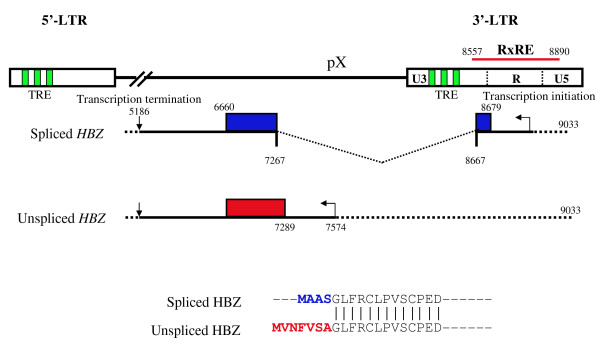
**Structure of spliced and unspliced *HBZ *genes**. The U5 and a part of R region of 3'LTR compose the promoter for the *HBZ *gene. The first exon of the spliced *HBZ *gene corresponds to the region that encodes the Rex responsive element (RxRE). The differences in the proteins derived from the spliced and the unspliced HBZ are 4 amino acids in the spliced HBZ and 7 amino acids in the unspliced HBZ.

Two transcripts have been reported that encode the *HBZ *gene (Figure 1); one is spliced (*sHBZ*) and the other is unspliced (*usHBZ*) [14,15]. The spliced transcript of the *HBZ *gene was first reported by Satou et al. [5], followed by subsequent reports that additionally identified a second minor spliced transcript [14,15]. Furthermore, while transcripts of the spliced *HBZ *gene were detected in all ATL cell lines and cells freshly isolated from ATL patients, the *tax *transcript was undetectable in some cell lines and most ATL cases [5]. Prior to this study, the transcription of HTLV-1 viral genes in ATL patients was deemed to be undetectable. Therefore, the *HBZ *gene is recognized as the first gene that is uniformly expressed in the leukemic cells of all ATL patients.

Both s*HBZ* and usHBZ have TATA-less promoters. sHBZ has multiple transcriptional initiation sites in the U5 and R regions of the 3' LTR, whereas the us*HBZ *gene initiates within the *tax *gene. It has been reported that Sp1 is critical for many TATA-less promoters. Consistent with this, the transcription of *sHBZ *gene is dependent on Sp1 [16].

Expression of the *sHBZ *gene which was detected not only in ATL cells but also in T-cells of asymptomatic carriers, appears to be correlated with provirus load [5]. Quantitative analyses of *HBZ *gene transcripts were reported by two groups [17,18]. The *sHBZ *gene transcripts were found to be four times higher than the *usHBZ *gene transcripts in both ATL patients and HTLV-1 carriers [17]. In addition, the half-life of the sHBZ protein isoform is longer than that of the usHBZ isoform [16]. Together, the data are consistent with Western blot analyses of HBZ protein in ATL cell lines that detected only sHBZ protein [19], further supporting the significance of sHBZ protein.

It has been reported that *HBZ *transcription is correlated with provirus load [17,18]. As described later, transcription of the *HBZ *gene is dependent on the basal transcription factor, Sp1 [16]. Therefore, it is conceivable that the *HBZ *transcripts are proportional to provirus load. More importantly, Saito *et al*. reported the correlation between the levels of *HBZ *gene transcripts and severity of HTLV-1 associated myelopathy/tropical spastic paraparesis (HAM/TSP), suggesting that *HBZ *gene expression might contribute to the pathogenesis of HAM/TSP [18].

### Structure of HBZ (the promoter, the coding genes, and the protein)

Anti-sense transcription of HTLV-1 was first reported in 1989 [20]. A decade later, the viral protein was detected in HTLV-1-transformed cell lines and further identified as a binding protein to CREB2 by the yeast two-hybrid method. This viral protein bound to CREB2 through its bZIP domain [21], and was designated as the HTLV-1 bZIP factor (HBZ). 5' rapid amplification of cDNA ends identified two different *HBZ *transcripts: spliced and unspliced forms (Figure 1) [5,14,15]. The promoter regions for the spliced and unspliced HBZ transcripts were identified, and both promoters are TATA-less. Sp1 has been identified as a critical transcription factor for the expression of the *sHBZ *gene [16]. The Tax-response element (TRE) motif, which is present in the U3 region of the LTR, functions as an enhancer of viral sense gene transcription. Tax forms a complex with CREB and p300/CBP, resulting in marked activation of viral gene transcription. The TRE in the 3' LTR also functions to enhance transcription of the *HBZ *antisense transcripts [16,22]. However, the enhancing activity for anti-sense transcription is relatively weak when compared with sense transcription [16]. This is consistent with the finding that transcription of the *HBZ *gene is relatively constant in ATL cases regardless of the variable expression levels of the *tax *gene [18].

**Figure 2:**
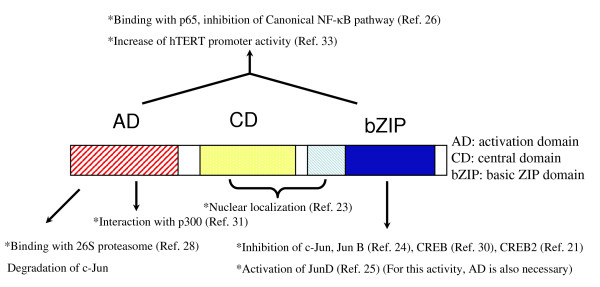
**Functional domains of HBZ protein**. HBZ has three domains: activation, central and bZIP domains. The interactions with host factors and the functions of HBZ are summarized in this Figure.

The HBZ protein contains three domains: activation, central and bZIP (Figure 2) [21,23]. HBZ binds to host factors with bZIP domains, which include c-Jun, JunB, JunD, CREB2 and CREB [24,25]. In addition, HBZ can bind to the p65 subunit of NF-κB [26]. The HBZ protein is localized in the nucleus with a speckled pattern [27]. Three regions are associated with nuclear localization: two regions rich in basic amino acids and a DNA binding domain (Figure 2). In addition, the integrity of the HBZ amino acid sequence is necessary for the speckled distribution in the nucleus. HBZ is localized in heterochromatin consistent with its association with transcriptional inhibition [23] In addition, HBZ has been shown to sequester JunB into nuclear bodies, thus suppressing JunB-dependent transcriptional activity [27].

The difference between the sHBZ and the usHBZ isoforms is only a few amino acids at the N-terminus (Figure 1) [15]. However, there are notably distinct characteristics. The half-life of sHBZ is much longer than that of usHBZ [16]. In addition, the sHBZ mRNA is more predominant than usHBZ mRNA [17]; thus, the protein level of sHBZ is much higher than that of usHBZ.

Growth-promoting activity was observed only in a T-cell line expressing sHBZ, but not in usHBZ-expressing T-cells [16]. Furthermore, *HBZ *RNA was shown to have growth promoting activity [5]. The difference between sHBZ and usHBZ lies with the presence of the first exon. This region corresponds to the Rex-responsive element (RxRE) in the R region of 3'LTR (Figure 1). RxRE forms a tight stem-loop structure, which is recognized by Rex to facilitate the nuclear export of viral RxRE-containing RNAs. The opposite strand of spliced HBZ RNA forms a different stem-loop structure, which might interact with host factors to induce the proliferation of ATL cells.

Biological differences between sHBZ and usHBZ proteins have also been demonstrated. usHBZ protein can induce the degradation of c-Jun in a ubiquitination-independent manner [28]. usHBZ protein directly interacts with both the 26 S proteasome and c-Jun, which results in the delivery of c-Jun to the proteasome. It has been reported that this activity of sHBZ is much weaker than that of usHBZ. However, inhibition of AP-1 mediated transcription by sHBZ was much stronger than that of usHBZ [16]. For the sHBZ protein, in addition to its higher protein level, its action to inhibit DNA binding by c-Jun or to sequester c-Jun in nuclear bodies might represent predominant mechanisms of transcriptional suppression.

### *In vitro *functions of HBZ

**Figure 3:**
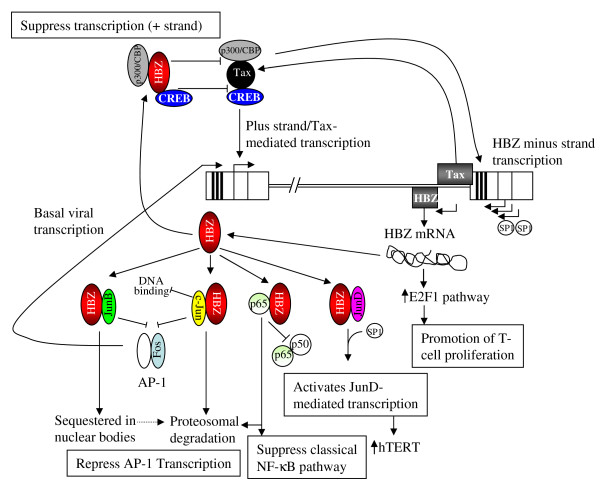
**Illustration of the expression and the activities of the HBZ RNA and protein**. Viral basal level plus-strand transcription is activated by AP-1 (Jun/Fos dimers) which initially favors Tax expression (hooked arrow denotes CAP site). Tax interacts with CREB and p300/CBP and the Tax-response element (TRE; 3 black bars in the viral promoter) to transactivate plus-strand transcription initially, leading to more Tax expression. Minus strand transcription of HBZ initiates (hooked arrows denote CAP sites) at multiple sites in the 3' LTR (sHBZ) and within the *tax *gene (usHBZ). sHBZ transcription is activated by SP1 with minor activation by Tax at the TRE in the 3'LTR. HBZ protein directly interacts with CREB and p300/CBP suppressing Tax-mediated plus-strand transcription. HBZ directly binds the Jun family members. Binding to JunB sequesters HBZ into nuclear bodies and may promote its proteosomal degradation. HBZ directly binds c-Jun, blocks its DNA binding activity, and facilitates its proteosomal degradation. HBZ binding of JunB and c-Jun prevents their interaction with Fos repressing both viral and cellular AP-1 transcription. HBZ directly interacts with JunD, and in conjunction with SP1 activates JunD-mediated transcription which includes the human telomerase reverse transcriptase gene (hTERT). HBZ also interacts with the p65 NFκB subunit, promotes its proteosomal degradation, and blocks its interaction with the NFκB p50 subunit resulting in the suppression of the classical NFκB transcriptional activation pathway. HBZ mRNA increases the expression of E2F1 which promotes T-lymphocyte proliferation.

*In vitro *studies investigating HBZ functions include both over-expression studies and those evaluating HBZ in the context of an infectious viral molecular clone. Initial studies utilized yeast two-hybrid analysis to show an interaction between HBZ bZIP binding domain and the bZIP transcription factor CREB2 (ATF-4) (see Figure 3). It was further shown that this interaction abolished the binding of CREB2 to the TRE in the HTLV-1 promoter and the cyclic AMP response element (CRE) in cellular promoters, consistent with the observations of HBZ dose-dependent down-regulation of Tax-mediated viral transcription [21,29]. Other cellular proteins including CREB and p300/CBP interact with HBZ and contribute to the down-regulation of Tax-dependent viral transcription [30,31]. However, the interaction of HBZ with p300/CBP is via two HBZ amino terminal motifs (not the HBZ bZIP domain) and the p300/CBP KIX domain [31]. HBZ, via its bZIP domain, also interacts with Jun family members including JunB, c-Jun, and JunD, thereby modulating their transcriptional activity [24,25]. Like CREB, HBZ decreases the DNA binding activity of JunB and c-Jun, thus disrupting basal transcription of both HTLV-1 and cellular promoters via attenuation of AP-1 activation (Fos/Jun dimers) [24,32]. Additional AP-1 transcriptional repression is explained by HBZ-mediated reduction in c-Jun stability via the proteasome-dependent pathway, and sequestration of JunB by HBZ within nuclear bodies [27,32]. In contrast to JunB and c-Jun, the interaction of HBZ with JunD stimulates its transcriptional activity and results in the activation of JunD-dependent cellular genes including human telomerase reverse transcriptase (hTERT) [33]. The significance of this finding is that the activation of telomerase is a critical late event in tumor progression and that HBZ is the only viral protein expressed in all ATL cells. Thus, the activation of telomerase by HBZ may contribute to the development and maintenance of leukemic cells.

It has been proposed that a highly regulated pattern of HTLV-1 gene expression is critical for virus-mediated T-lymphocyte immortalization/transformation and disease pathogenesis [34]. One study utilized real-time RT-PCR to determine the kinetics of viral gene expression in cells transiently transfected with an HTLV-1 proviral plasmid and in human T-lymphocytes newly infected by virus. The HTLV-1 gene expression profiles revealed that all sense and antisense transcripts increased over time and then plateaued to stable levels. *Gag/pol*, *tax/rex*, and *env *mRNAs were detected first and at the highest levels, whereas expression of the accessory genes, including the anti-sense *HBZ*, was at significantly lower levels than *tax/rex *[35]. Arnold *et al*. evaluated the functional role of HBZ in the context of an infectious molecular clone and, like other HTLV-1 accessory gene products, determined that the protein was dispensable for viral-induced immortalization of primary human T-lymphocytes [19]. However, a significant increase in p19 Gag production was observed in cell clones expressing HBZ defective proviruses, a finding consistent with the conclusion that in stable cell lines the loss of HBZ function results in increased Tax-mediated viral gene expression. Although the inhibition of Tax-mediated gene expression is a reported function of HBZ, the fact that HBZ is expressed in ATL cells lacking *tax *transcripts suggests that HBZ may have additional functions or activities. Satou *et al*. reported that repression of *HBZ *expression in ATL cell lines by shRNA resulted in a significant decrease in cell proliferation [5]. Moreover, shRNA repression of *HBZ *expression in established HTLV-1-transformed cell lines and newly immortalized T-lymphocytes also significantly suppressed T-lymphocyte proliferation [19]. Stable expression of HBZ enhanced IL-2-independent survival of Kit-225 and increased Jurkat cell proliferative capacity [[5] and Green unpublished]. Introduction of mutations that either abrogated HBZ protein expression or disrupted the HBZ mRNA without affecting the protein coding sequence indicated that the HBZ RNA, specifically a stem loop structure near the amino terminus of the gene, promoted T-cell proliferation; this contrasts with the finding that the HBZ protein inhibited Tax-mediated transactivation [5]. Thus, these findings led to the conclusion that the HBZ gene has a bimodal function in two different molecular forms. Microarray results and follow up analyses indicated that transcription of the *E2F1 *gene and its downstream targets were up-regulated in HBZ-expressing cells, suggesting a role for *E2F1 *in the enhanced proliferation mediated by HBZ [5]. The HBZ RNA, not the HBZ protein, was shown to be responsible for the up-regulation of *E2F1*.

In addition to binding transcription factors containing a bZIP domain, HBZ has been shown to bind the NF-κB subunit, p65, and inhibit NF-κB activation. Interestingly, this inhibition is selective for the classical NF-κB activation pathway, as the alternate NF-κB pathway is not inhibited [26]. Both the activation and the bZIP domains are important for the binding of HBZ with the Rel homology domain of p65 (Figure 2). HBZ not only inhibits DNA binding by p65, but also promotes the degradation of p65. As a mechanism of enhanced p65 degradation, HBZ increases the expression of E3 ubiquitin ligase, PDLIM2, resulting in ubiquitination and degradation of p65. Thus, HBZ can suppress the classical NF-κB pathway using two distinct mechanisms. Other viruses including EBV, African swine fever virus, hepatitis C virus, and human herpesvirus-8 have also been found to target p65 and inhibit the classical NF-κB pathway [36].

The classical and alternative NF-κB pathways have distinct regulatory functions. Accumulating evidence suggests that the alternative NF-κB pathway is more critical than the classical pathway in several cancers [37]. The two NF-κB pathways differentially control genes with anti-apoptotic functions in lymphoma cell lines [38]. Interestingly, HTLV-1 Tax is known to activate both the classical and the alternative NF-κB pathways, and it has been reported that the alternative pathway is critical to cellular transformation by Tax [39]. Potentially because HBZ selectively suppresses the classical NF-κB pathway, in cells that express both Tax and HBZ, Tax activity appears to predominantly activate NF-κB through the alternative pathway.

Recently, PDLIM2 has been reported to suppress Tax-mediated tumorigenicity by promoting degradation of Tax [40]. HBZ enhances the expression of PDLIM2 which should increase the degradation of Tax. Therefore, a complex scenario could exist in which HBZ suppresses Tax expression at the level of transcription and by enhanced degradation.

### *In vivo *functions of HBZ

HTLV-1 Tax plays a central role in the immortalization of T-lymphocytes in cell culture and in the early stages of leukemogenesis in infected patients. The observation that HBZ down-modulates Tax function while also promoting cellular proliferation suggests complex regulations that could influence viral latency, persistence, and disease. Although disruption of HBZ protein in an infectious proviral clone had no effect on the ability of the virus to immortalize T-lymphocytes in tissue culture, the loss of HBZ did result in a significant reduction of proviral load and an attenuated antibody response against viral proteins in a rabbit model of infection [29]. As early as two weeks post infection, the proviral load was reduced 5–50 fold, indicating that the HBZ protein was important very early in the infection process. Kinetic analysis of viral gene expression in PBMCs from newly infected rabbits revealed that *tax/rex *and *gag/pol *mRNAs were expressed at the highest levels immediately after infection and then progressively declined over time, eventually stabilizing at low levels [35]. Conversely, *HBZ *was expressed at a low level early after infection, and continued to increase before reaching a plateau, which was in direct correlation with proviral load levels in infected rabbit PBMCs. These results revealed an inverse correlation between *tax/rex *and *HBZ *mRNA expression over time, which provided important evidence linking *HBZ *expression to proviral load and the survival of the virus infected cell in an infected host.

Transgenic mice expressing HBZ under the control of the mouse CD4 promoter/enhancer displayed an increase in CD4^+ ^splenic T-lymphocytes suggesting that HBZ promotes proliferation of CD4^+ ^T-lymphocytes *in vivo *[5]. One transplant tumorigenicity study indicated that knockdown of *HBZ *in a transformed T-cell line significantly reduced tumor formation and organ infiltration in NOD/SCID^γchain-/- ^mice [19]. Taken together, these animal model studies further support a role for HBZ in the T-lymphocyte proliferation.

### The role of HBZ in HTLV-1 pathogenesis

The *HBZ *gene is expressed in ATL cells from all patients and its expression level is correlated with provirus load. In many ATL cases, *HBZ *is the only viral gene that is expressed. Furthermore, the *HBZ *gene has growth-promoting activity *in vivo *and *in vitro*, indicating that the *HBZ *gene likely is critical for ATL cells even at the late stage of leukemogenesis. There are two scenarios, not necessarily mutually exclusive, for the requirement of the *HBZ *gene in ATL induction. First, at the early stage of infection, both *tax *and *HBZ *genes are needed for the proliferation and maintenance of infected cells. Since Tax is the major target of CTL, cells without or with low Tax expression can evade immune surveillance. The finding that defective proviruses without a 5' LTR are generated after integration support this scenario. Second, only the *HBZ *gene is essential for maintaining oncogenesis while Tax contributes to the process that initiates and promotes cell growth with subsequently causes genetic instability. In either scenario, the *HBZ *gene is critical for oncogenesis by HTLV-1. In addition, since HBZ promotes proliferation of HTLV-1 infected T-cells, increased HTLV-1 infected cells should be implicated in pathogenesis of HAM/TSP. Since the *HBZ *gene is the sole viral gene expressed in ATL cells, it is an ideal target for therapy of not only ATL, but also HAM/TSP.

### Perspectives

Since the discovery of HTLV-1, there have been significant advances to our understanding of virus biology, immunology, and oncogenesis. However, the precise mechanism of oncogenesis by HTLV-1 remains to be determined. Recent intensive research on the antisense *HBZ *gene has yielded new important insight into the disease process: the *HBZ *gene appears to be the only viral gene that is constantly expressed in HTLV-1-infected cells and ATL cells. The data is consistent with HBZ playing a critical role in the proliferation of newly infected cells as well as in transformed ATL. Therefore, therapy targeted against HBZ might provide a promising new approach to the treatment of ATL as well as HAM/TSP. Some aspects of the HTLV-1 antisense transcript function may be conserved in other retroviruses since similar antisense RNAs have also been reported for HIV-1 [41].

## Competing interests

The authors declare that they have no competing interests.

## Authors' contributions

MM and PLG shared equally in the research, writing, and editing of the manuscript. Both authors read and approved the final manuscript.

## References

[B1] Takatsuki K (2005). Discovery of adult T-cell leukemia. Retrovirology.

[B2] Poiesz BJ, Ruscetti FW, Gazdar AF, Bunn PA, Minna JD, Gallo RC (1980). Detection and isolation of type C retrovirus particles from fresh and cultured lymphocytes of a patient with cutaneous T-cell lymphoma. Proc Natl Acad Sci USA.

[B3] Hinuma Y, Nagata K, Hanaoka M, Nakai M, Matsumoto T, Kinoshita K-I, Shirakawa S, Miyoshi I (1981). Adult T-cell leukemia: Antigen in an ATL cell line and detection of antibodies to the antigen in human sera. Proc Natl Acad Sci USA.

[B4] Takeda S, Maeda M, Morikawa S, Taniguchi Y, Yasunaga J, Nosaka K, Tanaka Y, Matsuoka M (2004). Genetic and epigenetic inactivation of tax gene in adult T-cell leukemia cells. Int J Cancer.

[B5] Satou Y, Yasunaga J, Yoshida M, Matsuoka M (2006). HTLV-I basic leucine zipper factor gene mRNA supports proliferation of adult T cell leukemia cells. Proc Natl Acad Sci USA.

[B6] Yoshida M (2001). Multiple viral strategies of HTLV-1 for dysregulation of cell growth control. Annu Rev Immunol.

[B7] Boxus M, Twizere JC, Legros S, Dewulf JF, Kettmann R, Willems L (2008). The HTLV-1 Tax interactome. Retrovirology.

[B8] Furukawa Y, Kubota R, Tara M, Izumo S, Osame M (2001). Existence of escape mutant in HTLV-I tax during the development of adult T-cell leukemia. Blood.

[B9] Tamiya S, Matsuoka M, Etoh K, Watanabe T, Kamihira S, Yamaguchi K, Takatsuki K (1996). Two types of defective human T-lymphotropic virus type I provirus in adult T-cell leukemia. Blood.

[B10] Miyazaki M, Yasunaga J, Taniguchi Y, Tamiya S, Nakahata T, Matsuoka M (2007). Preferential selection of human T-cell leukemia virus type 1 provirus lacking the 5' long terminal repeat during oncogenesis. J Virol.

[B11] Koiwa T, Hamano-Usami A, Ishida T, Okayama A, Yamaguchi K, Kamihira S, Watanabe T (2002). 5'-long terminal repeat-selective CpG methylation of latent human T-cell leukemia virus type 1 provirus in vitro and in vivo. J Virol.

[B12] Taniguchi Y, Nosaka K, Yasunaga J, Maeda M, Mueller N, Okayama A, Matsuoka M (2005). Silencing of human T-cell leukemia virus type I gene transcription by epigenetic mechanisms. Retrovirology.

[B13] Kannagi M, Harada S, Maruyama I, Inoko H, Igarashi H, Kuwashima G, Sato S, Morita M, Kidokoro M, Sugimoto M (1991). Predominant recognition of human T cell leukemia virus type I (HTLV-I) pX gene products by human CD8+ cytotoxic T cells directed against HTLV-I-infected cells. Int Immunol.

[B14] Cavanagh M-H, Landry S, Audet B, Arpin-Andre C, Hivin P, Pare M-E, Thete J, Wattel E, Marriott S, Mesnard J-M, Barbeau B (2006). HTLV-I antisense transcripts initiating in the 3' LTR are alternatively spliced and polyadenylated. Retrovirology.

[B15] Murata K, Hayashibara T, Sugahara K, Uemura A, Yamaguchi T, Harasawa H, Hasegawa H, Tsuruda K, Okazaki T, Koji T (2006). A novel alternative splicing isoform of human T-cell leukemia virus type 1 bZIP factor (HBZ-SI) targets distinct subnuclear localization. J Virol.

[B16] Yoshida M, Satou Y, Yasunaga J, Fujisawa J, Matsuoka M (2008). Transcriptional control of spliced and unspliced human T-cell leukemia virus type 1 bZIP factor (HBZ) gene. J Virol.

[B17] Usui T, Yanagihara K, Tsukasaki K, Murata K, Hasegawa H, Yamada Y, Kamihira S (2008). Characteristic expression of HTLV-1 basic zipper factor (HBZ) transcripts in HTLV-1 provirus-positive cells. Retrovirology.

[B18] Saito M, Matsuzaki T, Satou Y, Yasunaga J, Saito K, Arimura K, Matsuoka M, Ohara Y (2009). In vivo expression of the HBZ gene of HTLV-1 correlates with proviral load, inflammatory markers and disease severity in HTLV-1 associated myelopathy/tropical spastic paraparesis (HAM/TSP). Retrovirology.

[B19] Arnold J, Zimmerman B, Li M, Lairmore MD, Green PL (2008). Human T-cell Leukemia Virus Type-1 Antisense-encoded Gene, Hbz, Promotes T Lymphocyte Proliferation. Blood.

[B20] Larocca D, Chao LA, Seto MH, Brunck TK (1989). Human T-cell leukemia virus minus strand transcription in infected cells. Biochem Biophys Res Commun.

[B21] Gaudray G, Gachon F, Basbous J, Biard-Piechaczyk M, Devaux C, Mesnard J (2002). The complementary strand of the human T-cell leukemia virus type 1 RNA genome encodes a bZIP transcription factor that down-regulates viral transcription. J Virol.

[B22] Landry S, Halin M, Vargas A, Lemasson I, Mesnard JM, Barbeau B (2009). Upregulation of human T-cell leukemia virus type 1 antisense transcription by the viral tax protein. J Virol.

[B23] Hivin P, Frederic M, Arpin-Andre C, Basbous J, Gay B, Thebault S, Mesnard JM (2005). Nuclear localization of HTLV-I bZIP factor (HBZ) is mediated by three distinct motifs. J Cell Sci.

[B24] Basbous J, Arpin C, Gaudray G, Piechaczyk M, Devaux C, Mesnard J (2003). HBZ factor of HTLV-1 dimerizes with transcription factors JunB and c-Jun and modulates their transcriptional activity. J Biol Chem.

[B25] Thebault S, Basbous J, Hivin P, Devaux C, Mesnard JM (2004). HBZ interacts with JunD and stimulates its transcriptional activity. FEBS Lett.

[B26] Zhao T, Yasunaga J, Satou Y, Nakao M, Takahashi M, Fujii M, Matsuoka M (2009). Human T-cell leukemia virus type 1 bZIP factor selectively suppresses the classical pathway of NF-kappaB. Blood.

[B27] Hivin P, Basbous J, Raymond F, Henaff D, Arpin-Andre C, Robert-Hebmann V, Barbeau B, Mesnard JM (2007). The HBZ-SP1 isoform of human T-cell leukemia virus type I represses JunB activity by sequestration into nuclear bodies. Retrovirology.

[B28] Isono O, Ohshima T, Saeki Y, Matsumoto J, Hijikata M, Tanaka K, Shimotohno K (2008). Human T-cell leukemia virus type 1 HBZ protein bypasses the targeting function of ubiquitination. J Biol Chem.

[B29] Arnold J, Yamamoto B, Li M, Phipps AJ, Younis I, Lairmore MD, Green PL (2006). Enhancement of infectivity and persistence in vivo by HBZ, a natural antisense coded protein of HTLV-1. Blood.

[B30] Lemasson I, Lewis MR, Polakowski N, Hivin P, Cavanagh MH, Thebault S, Barbeau B, Nyborg JK, Mesnard JM (2007). Human T-cell leukemia virus type 1 (HTLV-1) bZIP protein interacts with the cellular transcription factor CREB to inhibit HTLV-1 transcription. J Virol.

[B31] Clerc I, Polakowski N, Andre-Arpin C, Cook P, Barbeau B, Mesnard JM, Lemasson I (2008). An interaction between the human T cell leukemia virus type 1 basic leucine zipper factor (HBZ) and the KIX domain of p300/CBP contributes to the down-regulation of tax-dependent viral transcription by HBZ. J Biol Chem.

[B32] Matsumoto J, Ohshima T, Isono O, Shimotohno K (2005). HTLV-1 HBZ suppresses AP-1 activity by impairing both the DNA-binding ability and the stability of c-Jun protein. Oncogene.

[B33] Kuhlmann AS, Villaudy J, Gazzolo L, Castellazzi M, Mesnard JM, Duc Dodon M (2007). HTLV-1 HBZ cooperates with JunD to enhance transcription of the human telomerase reverse transcriptase gene (hTERT). Retrovirology.

[B34] Li M, Green PL (2007). Detection and quantitation of HTLV-1 and HTLV-2 mRNA species by real-time RT-PCR. J Virol Methods.

[B35] Li M, Kesic M, Yin H, Lianbo Y, Green P (2009). Kinetic analysis of Human T-cell leukemia virus type 1 gene expression in cell culture and infected animals. J Virol.

[B36] Morrison TE, Kenney SC (2004). BZLF1, an Epstein-Barr virus immediate-early protein, induces p65 nuclear translocation while inhibiting p65 transcriptional function. Virology.

[B37] Keats JJ, Fonseca R, Chesi M, Schop R, Baker A, Chng WJ, Van Wier S, Tiedemann R, Shi CX, Sebag M (2007). Promiscuous mutations activate the noncanonical NF-kappaB pathway in multiple myeloma. Cancer Cell.

[B38] Bernal-Mizrachi L, Lovly CM, Ratner L (2006). The role of NF-{kappa}B-1 and NF-{kappa}B-2-mediated resistance to apoptosis in lymphomas. Proc Natl Acad Sci USA.

[B39] Higuchi M, Tsubata C, Kondo R, Yoshida S, Takahashi M, Oie M, Tanaka Y, Mahieux R, Matsuoka M, Fujii M (2007). Cooperation of NF-kappaB2/p100 activation and the PDZ domain binding motif signal in human T-cell leukemia virus type 1 (HTLV-1) Tax1 but not HTLV-2 Tax2 is crucial for interleukin-2-independent growth transformation of a T-cell line. J Virol.

[B40] Yan P, Fu J, Qu Z, Li S, Tanaka T, Grusby MJ, Xiao G (2009). PDLIM2 suppresses human T-cell leukemia virus type I Tax-mediated tumorigenesis by targeting Tax into the nuclear matrix for proteasomal degradation. Blood.

[B41] Landry S, Halin M, Lefort S, Audet B, Vaquero C, Mesnard JM, Barbeau B (2007). Detection, characterization and regulation of antisense transcripts in HIV-1. Retrovirology.

